# 1-[2-(2,4-Dichloro­phenyl)­pent­yl]-1*H*-1,2,4-triazole

**DOI:** 10.1107/S1600536809007120

**Published:** 2009-03-06

**Authors:** Corrado Rizzoli, Elda Marku, Lucedio Greci

**Affiliations:** a Dipartimento di Chimica Generale ed Inorganica, Chimica Analitica, Chimica Fisica, Universitá degli Studi di Parma, Viale G. P. Usberti 17/A, I-43100 Parma, Italy; b Fakulteti i Shkencave të Natyrës, Departamenti i Kimise, Universiteti i Tiranes, Bulevardi "Zogu I", Tirana, Albania; c Dipartimento ISAC, Universitá Politecnica delle Marche, Via Brecce Bianche, I-60131 Ancona, Italy

## Abstract

The title compound, C_13_H_15_Cl_2_N_3_, also known as penconazole, crystallizes as a racemate. The dihedral angle between the benzene and triazole rings is 24.96 (13)°. In the crystal structure, mol­ecules are linked into chains running parallel to the *c* axis by inter­molecular C—H⋯N hydrogen-bonding inter­actions.

## Related literature

For the synthesis and toxicity of the title compound, see: Maier *et al.* (1987 ▶); Worthing (1987 ▶); Tao *et al.* (2003 ▶). For the crystal structure of a related compound, see: Peeters *et al.* (1993 ▶).
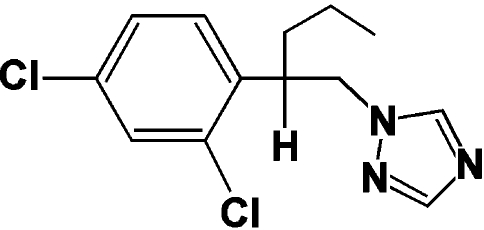


## Experimental

### 

#### Crystal data

C_13_H_15_Cl_2_N_3_
*M* *_r_* = 284.18
Monoclinic, 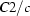

*a* = 25.083 (8) Å
*b* = 10.763 (2) Å
*c* = 11.206 (3) Å
β = 105.654 (3)°
*V* = 2913.1 (13) Å^3^
*Z* = 8
Cu *K*α radiation
μ = 3.89 mm^−1^
*T* = 297 K
0.23 × 0.20 × 0.16 mm

#### Data collection

Siemens AED diffractometer
Absorption correction: empirical (refined from Δ*F*) (*DIFABS*; Walker & Stuart, 1983 ▶) *T*_min_ = 0.432, *T*_max_ = 0.538
2737 measured reflections
2611 independent reflections
1183 reflections with *I* > 2σ(*I*)
*R*_int_ = 0.060
3 standard reflections every 100 reflections intensity decay: 0.01%

#### Refinement

*R*[*F* ^2^ > 2σ(*F* ^2^)] = 0.056
*wR*(*F* ^2^) = 0.127
*S* = 0.99
2611 reflections
163 parameters
H-atom parameters constrained
Δρ_max_ = 0.34 e Å^−3^
Δρ_min_ = −0.27 e Å^−3^

### 

Data collection: *AED* (Belletti *et al.*, 1993 ▶); cell refinement: *AED*; data reduction: *AED*; program(s) used to solve structure: *SHELXS97* (Sheldrick, 2008 ▶); program(s) used to refine structure: *SHELXL97* (Sheldrick, 2008 ▶); molecular graphics: *ORTEP-3 for Windows* (Farrugia, 1997 ▶) and *SCHAKAL* (Keller, 1997 ▶); software used to prepare material for publication: *SHELXL97* and *PARST95* (Nardelli, 1995 ▶).

## Supplementary Material

Crystal structure: contains datablocks global, I. DOI: 10.1107/S1600536809007120/hg2483sup1.cif

Structure factors: contains datablocks I. DOI: 10.1107/S1600536809007120/hg2483Isup2.hkl

Additional supplementary materials:  crystallographic information; 3D view; checkCIF report

## Figures and Tables

**Table 1:** Hydrogen-bond geometry (Å, °)

*D*—H⋯*A*	*D*—H	H⋯*A*	*D*⋯*A*	*D*—H⋯*A*
C3—H3*A*⋯N3^i^	0.97	2.52	3.489 (4)	174

Symmetry code: (i) 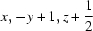.

**Fig. 1.**
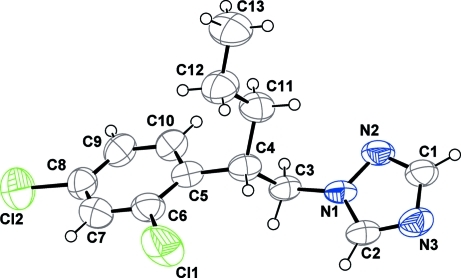
The molecular structure of the title compound. Displacement ellipsoids are drawn at the 50% probability level.

**Fig. 2.**
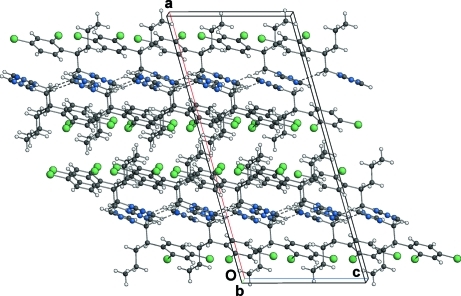
Crystal packing of the title compound viewed approximately along the b axis. Intermolecular C—H···N hydrogen bonds are shown as dashed lines.

**Fig. 3.**
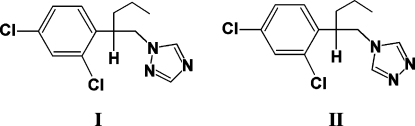
The structures of (I) and (II).

## supplementary crystallographic information

### Comment

The synthesis of the title compound, I, commonly known as penconazole, was described years ago (Maier *et al.*, 1987). Due to its ability to inhibit the development of fungi by interfering with sterol biosynthesis of their cell membranes, this product was introduced as an agriculture systemic fungicide affecting cucurbits, grapes, pome fruits and vegetables. The advantages of this compound is its low toxicity: acute oral dose (LD50) of 2125 mg/kg for rats (Worthing, 1987). More recently, penconazole was prepared by condensation of 2-(2,4-dichlorophenyl)-1-pentanole with 1,2,4-triazole (Tao *et al.*, 2003), but this method also leads to the formation of 1-(1*H*-1,3,4-triazol-1-yl)-2-(2,4-dichlorophenyl)-pentane (II) as a by-product. In repeating this reaction, our purpose was the determination of the crystal structure of the desired compound I and the evaluation of the percentage of the by-product II.

The title compound (Fig. 1) crystallizes as a racemate. The triazole ring is substantially planar (maximum deviation from planarity 0.006 (3) Å for atom C2) and forms a dihedral angle of 24.96 (13)° with the benzene ring. The N—N (1.351 (3) Å) and C—N (mean value 1.328 (4) Å) bond lengths within the triazole ring are comparable with those observed in 6-[(4-chlorophenyl)(1*H*-1,2,4-triazol-1-yl)methyl]-1-methyl-1*H*-benzotriazole (vorozole; Peeters *et al.*, 1993) and suggest electron delocalization over the ring. In the crystal structure, an intermolecular C—H···N hydrogen bonding interaction (Table 1) link the molecules into chains running parallel to the *c* axis (Fig. 2).

### Experimental

The title compound was prepared according to the literature reports (Tao *et al.*, 2003). This method afforded compounds I and II in a 93:3 ratio. The two compounds were separated by chromatography on SiO_2_ column eluting with cyclohexane/ethyl acetate (9:1 *v/v*). Crystals of the title compound suitable for X-ray analysis were obtained on slow evaporation of an *n*-pentane solution (m. p. 60–61°C). IR data, ν, cm^-1^: 3060, 1597, 1448, 760, 746, 700. ^1^H-NMR, δ in CDC*L*_3_: 0.87 (t, 3H, –CH2*CH3*), 1.23 (sextet, 2H, -*CH2*CH3), 2.6–2.8 (m,2*H*, –CH*CH2*CH2CH3), 3.78 (1*H*, quintet, –CH2*CH*CH2-), 4.34 (d, -*CH2*CH<), 7.23 (1*H*, speudo-q, H-5, J=8.3 Hz, J=2.2 Hz), 7.38 (1*H*, d, H-3, J=2.2 Hz), 7.71 (s, 1H, triazolyl-H-3), 7.89 (s,1*H*, triazolyl-H-5). MS, Calcd for C_13_H_15_Cl_2_N_3_, 284.2; Found. *M* (%): 250 (12.72), 248 (36.93), 161 (63.69), 159 (100); no molecular ion peak was observed; the highest peaks are those corresponding to the loss of a chlorine atom. The ^1^H-NMR spectrum of compound II shows a singlet at δ = 7.89 corresponding to the two equivalent H atoms of the 1,3,4-triazol-1-yl ring, the other part of the spectrum is strictly similar to that of compound I. Melting points were determined by an electrochemical apparatus and were uncorrected. ^1^H-NMR spectra were recorded on a Varian Gemini 200 MHz. IR spectra were recorded in the solid state with a Perkin-Elmer MGX1 spectrophotometer equipped with Spectra Tech. Mass spectra were recorded with a Carlo Erba QMD 1000 mass spectrometer in positive EI mode.

### Refinement

All H atoms were positioned geometrically with C—H = 0.93–0.98 Å, and refined using a riding model approximation with *U*_iso_(H) = 1.2 *U*_eq_(C) or 1.5 *U*_eq_(C) for methyl H atoms.

### Crystal data

**Table:**

C_13_H_15_Cl_2_N_3_	*F*(000) = 1184
*M**_r_* = 284.18	*D*_x_ = 1.296 Mg m^−^^3^
Monoclinic, *C*2/*c*	Cu *K*α radiation, λ = 1.54178 Å
Hall symbol: -C 2yc	Cell parameters from 48 reflections
*a* = 25.083 (8) Å	θ = 18.4–42.5°
*b* = 10.763 (2) Å	µ = 3.89 mm^−^^1^
*c* = 11.206 (3) Å	*T* = 297 K
β = 105.654 (3)°	Block, colourless
*V* = 2913.1 (13) Å^3^	0.23 × 0.20 × 0.16 mm
*Z* = 8	

### Data collection

**Table:**

Siemens AED diffractometer	1183 reflections with *I* > 2σ(*I*)
Radiation source: fine-focus sealed tube	*R*_int_ = 0.060
graphite	θ_max_ = 67.9°, θ_min_ = 3.7°
θ/2θ scans	*h* = −29→28
Absorption correction: empirical (using intensity measurements) (*DIFABS*; Walker & Stuart, 1983)	*k* = −2→12
*T*_min_ = 0.432, *T*_max_ = 0.538	*l* = −5→13
2737 measured reflections	3 standard reflections every 100 reflections
2611 independent reflections	intensity decay: 0.01%

### Refinement

**Table:**

Refinement on *F*^2^	Primary atom site location: structure-invariant direct methods
Least-squares matrix: full	Secondary atom site location: difference Fourier map
*R*[*F*^2^ > 2σ(*F*^2^)] = 0.056	Hydrogen site location: inferred from neighbouring sites
*wR*(*F*^2^) = 0.127	H-atom parameters constrained
*S* = 0.99	*w* = 1/[σ^2^(*F*_o_^2^) + (0.0456*P*)^2^] where *P* = (*F*_o_^2^ + 2*F*_c_^2^)/3
2611 reflections	(Δ/σ)_max_ < 0.001
163 parameters	Δρ_max_ = 0.34 e Å^−^^3^
0 restraints	Δρ_min_ = −0.27 e Å^−^^3^

### Special details

**Table:**

Refinement. Refinement of *F*^2^ against ALL reflections. The weighted *R*-factor *wR* and goodness of fit *S* are based on *F*^2^, conventional *R*-factors *R* are based on *F*, with *F* set to zero for negative *F*^2^. The threshold expression of *F*^2^ > σ(*F*^2^) is used only for calculating *R*-factors(gt) *etc*. and is not relevant to the choice of reflections for refinement. *R*-factors based on *F*^2^ are statistically about twice as large as those based on *F*, and *R*- factors based on ALL data will be even larger.

### Fractional atomic coordinates and isotropic or equivalent isotropic displacement parameters (Å^2^)

**Table:**

	*x*	*y*	*z*	*U*_iso_*/*U*_eq_	
Cl1	0.08070 (6)	0.51687 (9)	0.93474 (12)	0.1422 (6)	
Cl2	0.08744 (5)	0.18168 (11)	1.28987 (10)	0.1305 (5)	
N1	0.22774 (10)	0.3780 (2)	0.7443 (2)	0.0595 (7)	
N2	0.24356 (12)	0.3032 (2)	0.6633 (2)	0.0757 (8)	
N3	0.25582 (12)	0.5043 (2)	0.6210 (3)	0.0815 (9)	
C1	0.25951 (15)	0.3839 (3)	0.5932 (3)	0.0808 (10)	
H1	0.2728	0.3591	0.5270	0.097*	
C2	0.23549 (13)	0.4960 (3)	0.7196 (3)	0.0714 (9)	
H2	0.2279	0.5634	0.7644	0.086*	
C3	0.20829 (13)	0.3285 (3)	0.8460 (3)	0.0638 (8)	
H3A	0.2230	0.3790	0.9192	0.077*	
H3B	0.2226	0.2449	0.8646	0.077*	
C4	0.14554 (13)	0.3253 (3)	0.8180 (3)	0.0666 (8)	
H4	0.1313	0.4086	0.7921	0.080*	
C5	0.13060 (12)	0.2927 (3)	0.9380 (3)	0.0648 (8)	
C6	0.10164 (15)	0.3713 (3)	0.9946 (3)	0.0807 (10)	
C7	0.08852 (16)	0.3387 (3)	1.1047 (4)	0.0927 (11)	
H7	0.0695	0.3937	1.1424	0.111*	
C8	0.10441 (15)	0.2235 (4)	1.1558 (3)	0.0790 (10)	
C9	0.13257 (14)	0.1450 (3)	1.1013 (3)	0.0787 (10)	
H9	0.1435	0.0680	1.1372	0.094*	
C10	0.14543 (13)	0.1774 (3)	0.9930 (3)	0.0724 (9)	
H10	0.1643	0.1211	0.9562	0.087*	
C11	0.11930 (14)	0.2323 (3)	0.7113 (3)	0.0830 (10)	
H11A	0.1315	0.2537	0.6389	0.100*	
H11B	0.1326	0.1492	0.7368	0.100*	
C12	0.05814 (16)	0.2321 (4)	0.6773 (3)	0.1057 (13)	
H12A	0.0450	0.3152	0.6514	0.127*	
H12B	0.0460	0.2115	0.7501	0.127*	
C13	0.03234 (16)	0.1433 (4)	0.5764 (4)	0.1191 (15)	
H131	−0.0072	0.1482	0.5589	0.179*	
H132	0.0442	0.0604	0.6020	0.179*	
H133	0.0434	0.1640	0.5032	0.179*	

### Atomic displacement parameters (Å^2^)

**Table:**

	*U*^11^	*U*^22^	*U*^33^	*U*^12^	*U*^13^	*U*^23^
Cl1	0.2339 (15)	0.0687 (6)	0.1461 (10)	0.0447 (8)	0.0895 (10)	0.0118 (7)
Cl2	0.1620 (11)	0.1516 (11)	0.0905 (7)	−0.0169 (8)	0.0558 (7)	0.0066 (7)
N1	0.0728 (18)	0.0384 (12)	0.0602 (15)	−0.0030 (12)	0.0061 (13)	−0.0050 (12)
N2	0.103 (2)	0.0486 (14)	0.0760 (18)	−0.0110 (15)	0.0248 (16)	−0.0087 (14)
N3	0.101 (2)	0.0588 (17)	0.081 (2)	−0.0131 (15)	0.0183 (18)	0.0088 (15)
C1	0.111 (3)	0.0566 (19)	0.075 (2)	−0.016 (2)	0.026 (2)	−0.0020 (18)
C2	0.084 (3)	0.0447 (17)	0.079 (2)	−0.0054 (16)	0.0105 (19)	−0.0048 (17)
C3	0.076 (2)	0.0485 (16)	0.0612 (19)	−0.0073 (15)	0.0083 (16)	0.0029 (15)
C4	0.072 (2)	0.0545 (18)	0.067 (2)	0.0001 (16)	0.0072 (17)	0.0065 (16)
C5	0.064 (2)	0.0564 (18)	0.066 (2)	−0.0038 (16)	0.0049 (16)	−0.0013 (16)
C6	0.105 (3)	0.057 (2)	0.082 (2)	−0.003 (2)	0.027 (2)	−0.0030 (19)
C7	0.109 (3)	0.079 (3)	0.094 (3)	−0.006 (2)	0.035 (2)	−0.019 (2)
C8	0.086 (3)	0.089 (3)	0.064 (2)	−0.015 (2)	0.0244 (19)	0.002 (2)
C9	0.079 (2)	0.074 (2)	0.079 (2)	−0.0020 (19)	0.014 (2)	0.019 (2)
C10	0.073 (2)	0.066 (2)	0.076 (2)	0.0035 (17)	0.0154 (18)	0.0082 (18)
C11	0.084 (3)	0.094 (3)	0.060 (2)	−0.011 (2)	0.0009 (18)	−0.0074 (19)
C12	0.096 (3)	0.121 (3)	0.091 (3)	−0.026 (3)	0.011 (2)	−0.011 (3)
C13	0.095 (3)	0.140 (4)	0.100 (3)	−0.016 (3)	−0.013 (2)	−0.030 (3)

### Geometric parameters (Å, °)

**Table:**

Cl1—C6	1.729 (3)	C5—C10	1.391 (4)
Cl2—C8	1.728 (3)	C6—C7	1.404 (5)
N1—C2	1.326 (3)	C7—C8	1.379 (4)
N1—N2	1.351 (3)	C7—H7	0.9300
N1—C3	1.457 (3)	C8—C9	1.348 (4)
N2—C1	1.304 (4)	C9—C10	1.382 (4)
N3—C2	1.339 (4)	C9—H9	0.9300
N3—C1	1.342 (4)	C10—H10	0.9300
C1—H1	0.9300	C11—C12	1.478 (4)
C2—H2	0.9300	C11—H11A	0.9700
C3—C4	1.520 (4)	C11—H11B	0.9700
C3—H3A	0.9700	C12—C13	1.488 (5)
C3—H3B	0.9700	C12—H12A	0.9700
C4—C5	1.530 (4)	C12—H12B	0.9700
C4—C11	1.562 (4)	C13—H131	0.9600
C4—H4	0.9800	C13—H132	0.9600
C5—C6	1.377 (4)	C13—H133	0.9600
			
C2—N1—N2	110.2 (3)	C8—C7—H7	120.7
C2—N1—C3	127.8 (3)	C6—C7—H7	120.7
N2—N1—C3	122.0 (2)	C9—C8—C7	120.2 (3)
C1—N2—N1	101.6 (2)	C9—C8—Cl2	120.9 (3)
C2—N3—C1	101.1 (3)	C7—C8—Cl2	118.9 (3)
N2—C1—N3	116.8 (3)	C8—C9—C10	121.0 (3)
N2—C1—H1	121.6	C8—C9—H9	119.5
N3—C1—H1	121.6	C10—C9—H9	119.5
N1—C2—N3	110.2 (3)	C9—C10—C5	121.1 (3)
N1—C2—H2	124.9	C9—C10—H10	119.5
N3—C2—H2	124.9	C5—C10—H10	119.5
N1—C3—C4	113.2 (2)	C12—C11—C4	113.1 (3)
N1—C3—H3A	108.9	C12—C11—H11A	109.0
C4—C3—H3A	108.9	C4—C11—H11A	109.0
N1—C3—H3B	108.9	C12—C11—H11B	109.0
C4—C3—H3B	108.9	C4—C11—H11B	109.0
H3A—C3—H3B	107.8	H11A—C11—H11B	107.8
C3—C4—C5	108.0 (2)	C11—C12—C13	113.9 (3)
C3—C4—C11	111.8 (3)	C11—C12—H12A	108.8
C5—C4—C11	111.9 (2)	C13—C12—H12A	108.8
C3—C4—H4	108.3	C11—C12—H12B	108.8
C5—C4—H4	108.3	C13—C12—H12B	108.8
C11—C4—H4	108.3	H12A—C12—H12B	107.7
C6—C5—C10	117.1 (3)	C12—C13—H131	109.5
C6—C5—C4	123.3 (3)	C12—C13—H132	109.5
C10—C5—C4	119.6 (3)	H131—C13—H132	109.5
C5—C6—C7	122.0 (3)	C12—C13—H133	109.5
C5—C6—Cl1	121.4 (3)	H131—C13—H133	109.5
C7—C6—Cl1	116.7 (3)	H132—C13—H133	109.5
C8—C7—C6	118.7 (3)		

### Hydrogen-bond geometry (Å, °)

**Table:**

*D*—H···*A*	*D*—H	H···*A*	*D*···*A*	*D*—H···*A*
C3—H3A···N3^i^	0.97	2.52	3.489 (4)	174

Symmetry codes: (i) *x*, −*y*+1, *z*+1/2.
